# Quantification of Cytokine Storms During Virus Infections

**DOI:** 10.3389/fimmu.2021.659419

**Published:** 2021-05-17

**Authors:** Shu Yuan, Si-Cong Jiang, Zhong-Wei Zhang, Yu-Fan Fu, Jing Hu, Zi-Lin Li

**Affiliations:** ^1^ College of Resources, Sichuan Agricultural University, Chengdu, China; ^2^ Chengdu KangHong Pharmaceutical Group Comp. Ltd., Chengdu, China; ^3^ School of Medicine, Northwest University, Xi’an, China; ^4^ Department of Cardiovascular Surgery, Xijing Hospital, Medical University of the Air Force, Xi’an, China

**Keywords:** cytokine storm, IL-4, IFN-γ, viral load, clearance rate

## Abstract

Highly pathogenic virus infections usually trigger cytokine storms, which may have adverse effects on vital organs and result in high mortalities. The two cytokines interleukin (IL)-4 and interferon (IFN)-γ play key roles in the generation and regulation of cytokine storms. However, it is still unclear whether the cytokine with the largest induction amplitude is the same under different virus infections. It is unknown which is the most critical and whether there are any mathematical formulas that can fit the changing rules of cytokines. Three coronaviruses (SARS-CoV, MERS-CoV, and SARS-CoV-2), three influenza viruses (2009H1N1, H5N1 and H7N9), Ebola virus, human immunodeficiency virus, dengue virus, Zika virus, West Nile virus, hepatitis B virus, hepatitis C virus, and enterovirus 71 were included in this analysis. We retrieved the cytokine fold change (FC), viral load, and clearance rate data from these highly pathogenic virus infections in humans and analyzed the correlations among them. Our analysis showed that interferon-inducible protein (IP)-10, IL-6, IL-8 and IL-17 are the most common cytokines with the largest induction amplitudes. Equations were obtained: the maximum induced cytokine (max) FC = IFN-γ FC × (IFN-γ FC/IL-4 FC) (if IFN-γ FC/IL-4 FC > 1); max FC = IL-4 FC (if IFN-γ FC/IL-4 FC < 1). For IFN-γ-inducible infections, 1.30 × log2 (IFN-γ FC) = log10 (viral load) − 2.48 − 2.83 × (clearance rate). The clinical relevance of cytokines and their antagonists is also discussed.

## Introduction

A large number of studies have demonstrated that viral infections can cause multiple complications in patients that result in multi-organ failure. These may be related to hyper-immune responses to the viruses and may have adverse effects on vital organs and lead to high pathogenicity and mortality (1–4). For example, the H5N1 influenza virus sets off a cytokine storm, including but not limited to interferon (IFN)-β, interleukin (IL)-6, and interferon-inducible protein (IP)-10 (1–4), and SARS patients showed increased amounts of proinflammatory cytokines in serum [e.g., IL-1b, IL-6, IL-12, IFN-γ, IP-10, and monocyte chemotactic protein (MCP)-1], which are associated with pulmonary inflammation and extensive lung damage (5). MERS infection was also reported to induce increased levels of proinflammatory cytokines (e.g., IFN-γ, tumor necrosis factor (TNF)-α, IL-15, and IL-17) (6). However, it is still unclear whether the cytokine with the largest induction amplitude is the same under different virus infections. It is unknown which among these cytokines is the most critical and whether there are any mathematical models that can fit the dynamics of the cytokines.

## Materials and Methods

### Data Extraction and Quality Assessment

We conducted a literature search of peer-reviewed publications in the PubMed electronic database from its inception to October 15, 2020. Only human infections by highly pathogenic viruses were included in this analysis. They were three coronaviruses (SARS-CoV, MERS-CoV, and SARS-CoV-2), three influenza viruses (2009H1N1, H5N1 and H7N9), Ebola virus, human immunodeficiency virus, dengue virus, Zika virus, West Nile virus, hepatitis B virus, hepatitis C virus, and enterovirus 71. Low-pathogenic seasonal H3N2 infection was investigated as the control group.

To get cytokine change information, the following three sets of keywords were employed for the literature search: “patient” (keyword 1), “cytokine” (keyword 2) and individual virus name (keyword 3) (**Figure S1**). We retrieved the full text of the potentially eligible studies and examined the full-text reports to obtain information about fold changes of cytokines relative to the healthy (or convalescent) control group. The articles without individual cytokine levels in the control group were excluded. Studies where n < 5 (such as some case reports) or three or more FC > 100 (which should be outliers) were also excluded because that the data may be unreliable. If there were multiple sampling time points, the highest (peak) value was selected. For the case where two or more references showed the same cytokines, the data with the largest n value were selected. Some cytokine levels in the control group were extremely low (close to zero) and were therefore excluded from subsequent analysis or adjusted to the normal levels as indicated in the references (**Table S1**). If none of these articles included either IFN-γ or IL-4, then more articles that included these two cytokines were searched (keyword 2 “patient” was replaced with “IL-4” or “IFN-γ”). The final set of papers with their cytokine information are listed in **Tables S1** and **S2**.

To get viral load information, the following three sets of keywords were employed for the literature search: “patient” (keyword 1), “viral load” (keyword 2) and individual virus name (keyword 3) (**Figure S2**). Different reports presented viral loads in different ways, such as cycle threshold (Ct) values of the RT-PCR analysis, log_10_ viral RNA copies, or absolute viral titers. Only the data expressed as RNA copies/mL were recorded. The papers where n < 5 (such as some case reports) were excluded. For respiratory viruses, viral loads in throat swabs, sputum, or lower respiratory tracts were recorded. For the other viruses, serum viral loads were recorded. If there were multiple sampling sites, the highest value was selected; if there were multiple sampling time points, the highest (peak) value was selected. In case two or more references showed the same cytokines, the data with the largest n value were selected (**Table S3**).

To obtain virus clearance time information, the following three sets of keywords were employed for the literature search: “patient” (keyword 1), “viral shedding or clearance” (keyword 2) and individual virus name (keyword 3) (**Figure S3**). The duration of viral shedding or the time from onset of symptoms to negative PCR result (50^th^ percentiles for the time until the loss of virus RNA detection) for each virus was recorded. The papers where n < 5 (such as some case reports) or only with the time of hospital stay or the duration of fever were excluded. For respiratory viruses, median durations of virus in respiratory samples were recorded. For other viruses, data in serum samples were recorded. In case two or more references showed the viral load or the clearance time from the same infection, the data with the largest n value were selected (**Table S3**).

We did not consider the deviation effects of drug treatments because for a certain virus infection with a certain symptom, the drug treatment is usually fixed. For example, both oseltamivir and antibiotics are usually given for severe influenza virus infections (independent of hemagglutinin types) at the time of admission (7). Cytokine data, viral load, and clearance rate were usually acquired from the same patients with the same drug treatment.

### Data Processing and Statistical Analysis

Correlations between IFN-γ FC and max FC, IL-4 FC and max FC, [IFN-γ FC × (IFN-γ FC/IL-4 FC) (if IFN-γ FC/IL-4 FC > 1)] or [IFN-γ FC × (IL-4 FC/IFN-γ FC) (if IFN-γ FC/IL-4 FC < 1)] and max FC were calculated.

Correlations between viral load and log_2_ (IFN-γ FC) or between clearance rate and log_2_ (IFN-γ FC) were calculated. For Ebola survivors, Ebola fatalities, dengue hemorrhagic fever patients, and HCV patients, the clearance rates were close to zero (**Table S3**). Under these four conditions, the IFN-γ levels may be mainly affected by the viral loads and not by the clearance rates. We calculated the linear regression between IFN-γ FC and log_10_ (viral load), log_2_ (IFN-γ FC) and log_10_ (viral load), or log_10_ (IFN-γ FC) and the log_10_ (viral load). Log_2_ (IFN-γ FC) got the best linear regression: log_10_ (viral load) – 2.48 = 1.30 × log_2_ (IFN-γ FC).

Given that viral load and clearance rate are positively and negatively correlated with IFN-γ FC, respectively, we presume that 1.30 × log_2_ (IFN-γ FC) = log_10_ (viral load) – 2.48 − coefficient × clearance rate. Among all the viruses selected in this study, the fastest clearance happens to 2009H1N1 that 6.84 log_10_ virus particles (8) could be cleared within 5 days in the mild patients (9) with only a 1.3 fold-induction to IFN-γ (10); thus the coefficient could be calculated as 2.83. Then, the correlation between [log_10_ (viral load) − 2.48 − 2.83 × (clearance rate)]/1.30 and log_2_ (IFN-γ FC) was calculated.

The F-test was performed to analyze all the correlations and determine whether the data pairs fit the regression model. The regression equation, the correlation coefficient, and the P-value were obtained by using SPSS v19.0 and Microsoft Excel 2013. P-value threshold and R^2^ threshold for statistical significance for claims of correlations were 0.05 and 0.5 respectively.

## Results

### IP-10, L-6, IL-8, and IL-17 Are the Most Increased Cytokines

**Figure 1:**
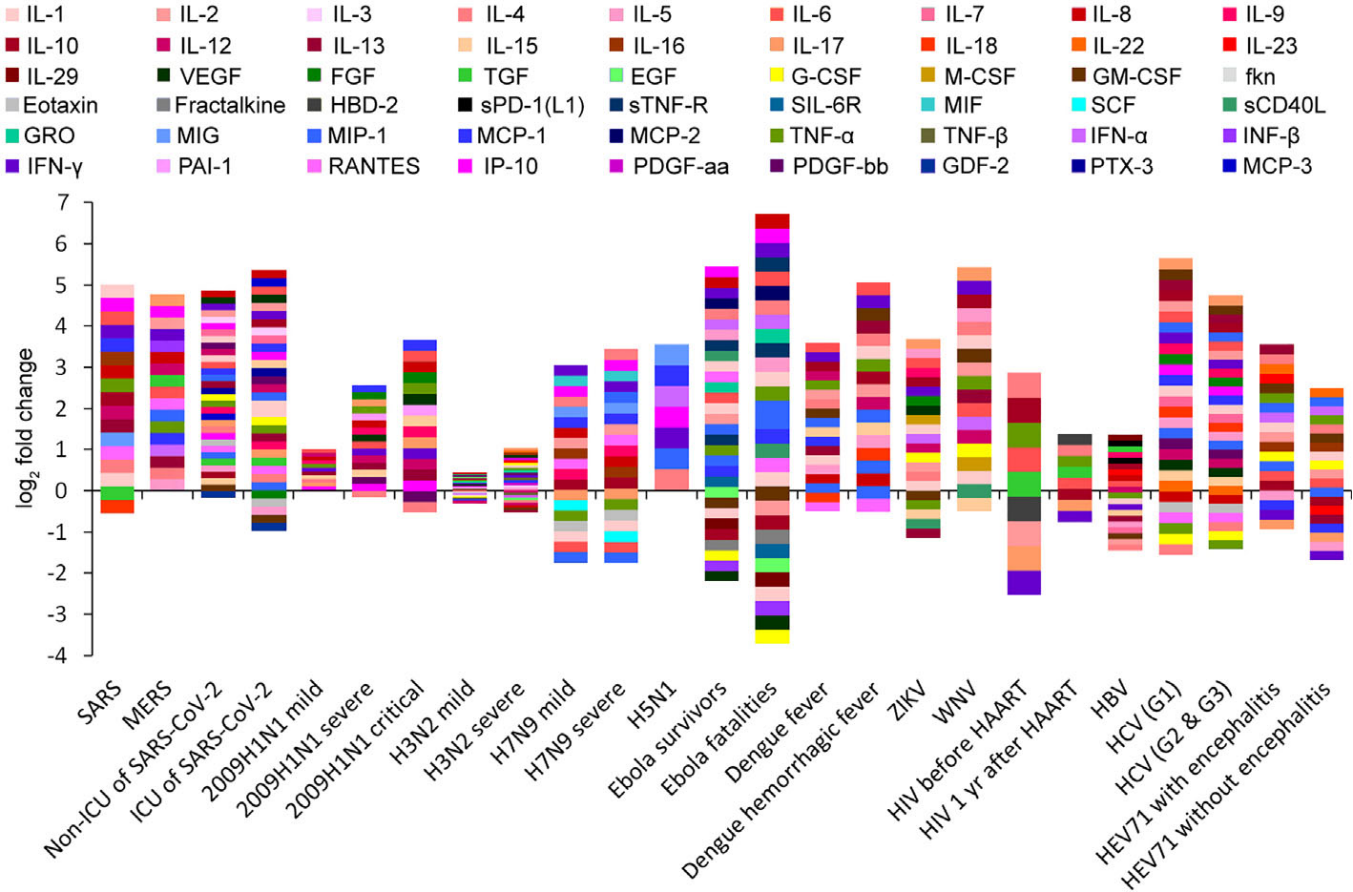
Cytokine profiling barcodes during different virus infections. ICU, intensive care unit; HAART, highly active antiretroviral therapy.

We conducted a literature search of peer-reviewed publications in the PubMed electronic database and retrieved the cytokine fold change (FC), viral load, and clearance rate data from highly pathogenic virus infections in humans for three coronaviruses (SARS-CoV, MERS-CoV, and SARS-CoV-2), three influenza viruses (2009H1N1, H5N1, and H7N9), Ebola virus (EBOV), human immunodeficiency virus (HIV), dengue virus (DENV), Zika virus (ZIKV), West Nile virus (WNV), hepatitis B virus (HBV), hepatitis C virus (HCV), and enterovirus 71 (EV71). Low-pathogenic seasonal H3N2 infection was used as a control sample (**Figures S1–S3** and **Tables S1–S4**). According to the severity of symptoms, a total of 25 virus infection cases were summarized, and the two cytokines with the most significant increases were recorded in each case (**Figures 1**, **S4–S8**). Most of the cytokines increased after viral infections, with a maximum increase of 102 times, and the increase in some individual patients was up to 200 times (e.g., IL-8 in Ebola fatalities; **Figure S6**) (11). However, some other cytokines were significantly inhibited by viral infections, such as granulocyte colony-stimulating factor (G-CSF) and vascular endothelial growth factor (VEGF) in Ebola virus infection (**Figures 1**, **S6**) (11) and IFN-γ and IL-17 in HIV infection (**Figures 1**, **S8**) (12). Among the 50 cytokines with the largest induction amplitudes, IP-10, L-6, IL-8, and IL-17 appeared with the highest frequency (5 out of 50). IFN-γ and IL-4 appeared four times; and MCP-1 appeared three times (**Table S2**). It is worth noting that the cytokines with the largest induction amplitudes were not necessarily the same in mild patients and severe patients with the same virus infection. For example, in EV71 patients with encephalitis, IL-13 and IL-4 were the most increased cytokines, whereas in EV71 patients without encephalitis, IL-22 and macrophage inflammatory protein-1a (MIP-1a) were the most increased cytokines (**Table S2** and **Figure S8**) (13, 14).

### Variations of IFN-γ and IL-4 Determine the Maximum Amplitude of All Cytokines

**Figure 2:**
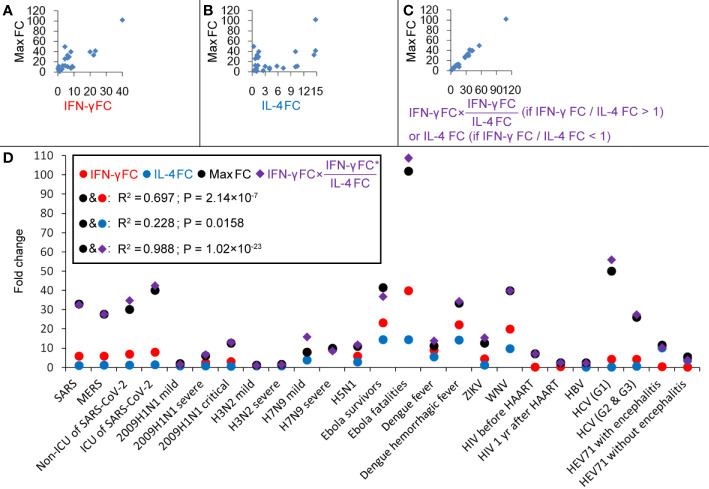
Correlations between cytokines during different virus infections. **(A)** Correlation between IFN-γ fold-changes (FC) and the maximum induced cytokine (max) FC. **(B)** Correlation between IL-4 FC and max FC. **(C)** Correlation between [IFN-γ FC × (IFN-γ FC/IL-4 FC) (if IFN-γ FC/IL-4 FC > 1)] or [IFN-γ FC × (IL-4 FC/IFN-γ FC) (if IFN-γ FC/IL-4 FC < 1)] and max FC. **(D)** Fold-changes of IFN-γ, IL-4 and max and values of [IFN-γ FC × (IFN-γ FC/IL-4 FC) (if IFN-γ FC/IL-4 FC > 1)] or [IFN-γ FC × (IL-4 FC/IFN-γ FC) (if IFN-γ FC/IL-4 FC < 1)].

Cytokines produced by T helper (Th) cells are of critical importance for the outcome of many infectious diseases. Producing the “right” set of cytokines in response to an infectious agent can be a matter of life or death. Although the Thl/Th2 dichotomy (mutual antagonistic loop) is an oversimplification, it has proven useful in the analysis of immune responses to infections (Infante-Duart and Kamradt, 1999; Paludan et al., 1998). The two cytokines IL-4 and IFN-γ play major roles in the generation and regulation of immune responses. Central in this respect are their mutually antagonistic functions. IFN-γ plays a key role in the inhibition of Th2-cell differentiation and Th1-cell stabilization; IL-4 promotes Th2-cell differentiation and stability and inhibits Th1-cell differentiation (15, 16). A significant correlation between IFN-γ FC and the maximum induced cytokine (max) FC was found (R^2^ = 0.697; **Figure 2A**), and no significant correlation between IL-4 FC and max FC was found (R^2^ = 0.228; **Figure 2B**). Thus, IFN-γ may determine the fluctuation amplitude of cytokines in innate immune responses, and this input amplitude is amplified by the Th1/Th2 core oscillator in the adaptive immune responses. The amplification factor should be the ratio of IFN-γ FC/IL-4 FC (if IFN-γ FC/IL-4 FC < 1, the amplification factor should be the ratio of IL-4 FC/IFN-γ FC). So the product of IFN-γ FC and the amplification factor may present the overall magnification of cytokine-inducing signals. Correlation between [IFN-γ FC × (IFN-γ FC/IL-4 FC) (if IFN-γ FC/IL-4 FC > 1)] or [IFN-γ FC × (IL-4 FC/IFN-γ FC) (if IFN-γ FC/IL-4 FC < 1)] and the max FC was calculated and a very high correlation coefficient R^2^ = 0.988 was obtained (**Figures 2C, D**), indicating that this formula can very well predict the maximum amplitude of cytokines. For the condition that IFN-γ FC/IL-4 FC > 1 (Th1-type infections), max FC = (IFN-γ FC)^2^/IL-4 FC, which means that every two times of IFN-γ increase would result in a maximum four times increase of all cytokines. For the condition that IFN-γ FC/IL-4 FC < 1 (Th2-type infections, such as HIV and EV71 infections), max FC = IFN-γ FC × (IL-4 FC/IFN-γ FC) = IL-4 FC, which means that the range of variation of IL-4 is the maximum amplitude of all cytokines.

### Viral Load and Clearance Rate Are Positively and Negatively Correlated With IFN-γ Fold-Changes

**Figure 3:**
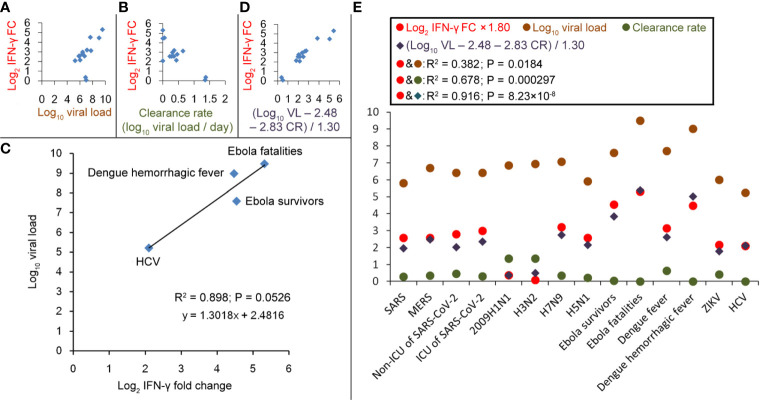
Correlations between IFN-γ fold-changes (FC) and viral load or virus clearance rate during different virus infections. **(A)** Correlation between log_10_ (viral load) and log_2_ (IFN-γ FC). **(B)** Correlation between virus clearance rate and log_2_ (IFN-γ FC). **(C)** Linear regression between log_10_ (viral load) and log_2_ (IFN-γ FC) in patients with EBOV, HCV or Dengue hemorrhagic fever. **(D)** Correlation between [log_10_ (viral load; VL) − 2.48 − 2.83 × (clearance rate; CR)]/1.30 and log_2_ (IFN-γ FC). **(E)** Values of log_2_ (IFN-γ FC), log_10_ (viral load), clearance rate and (log_10_ VL − 2.48 − 2.83 × CR)/1.30.

Next, the determinants of IFN-γ changes were investigated. For the condition that IFN-γ increased after the infection, viral load was weakly positive-correlated with log_2_ (IFN-γ FC) (R^2^ = 0.382; **Figure 3A**), and virus clearance rate was negatively correlated with log_2_ (IFN-γ FC) (R^2^ = 0.678; **Figure 3B**). Ebola survivors need 158 days to clear the virus, and Ebola fatalities result when the individual cannot clear the virus before death (17). Patients with primary dengue hemorrhagic fever need a long time (far more than 12 days) to clear the viral protein NS1 (18); 85% of HCV patients cannot clear the virus within 9 months (15% of patients cleared HCV spontaneously within 108 days) (19, 20). Their clearance rates are close to zero. Thus, under these four conditions, the IFN-γ levels may be mainly affected by the viral loads but not by the clearance rates. Then, we calculated the linear regression between IFN-γ FC and log_10_ (viral load), log_2_ (IFN-γ FC) and log_10_ (viral load), or log_10_ (IFN-γ FC) and the log_10_ (viral load). Log_2_ (IFN-γ FC) got the best linear regression (**Figure 3C**). The following regression equation was obtained: log_10_ (viral load) – 2.48 = 1.30 × log_2_ (IFN-γ FC), which means that every 10-fold increase in viral load would result in 1.7-fold induction to IFN-γ. The intercept of 2.48 means that when viral load < 2.48 log_10_, IFN-γ could not be enhanced by the infection. In fact, viral loads less than 10^2^-10^3^ are usually below the PCR detection limit and do not induce cytokine storms (17–21).

Given that viral load and clearance rate are positively and negatively correlated with IFN-γ FC, respectively, we presume that 1.30 × log_2_ (IFN-γ FC) = log_10_ (viral load) – 2.48 − coefficient × clearance rate. Among all the viruses selected in this study, the fastest clearance happens to 2009H1N1 that 6.84 log_10_ virus particles (8) could be cleared within 5 days in the mild patients (9) with only a 1.3 fold-induction to IFN-γ (10); the coefficient could be calculated as 2.83. For the IFN-γ-inducible conditions, the correlation between [log_10_ (viral load) − 2.48 − 2.83 × (clearance rate)]/1.30 and log_2_ (IFN-γ FC) was calculated and a high correlation coefficient R^2^ = 0.916 was obtained (**Figures 3D, E**), which suggests that the inducing amplitude of IFN-γ could be predicted according to the viral load and the clearance rate. However, no such correlations could be found for the condition that IFN-γ decreased after the infection (such as HIV, HBV, and EV71 infections).

## Discussion

The cytokines with the largest induction amplitudes are summarized in this study. Their antagonists may be used clinically to control the inflammatory responses. However, under different virus infections, the most significantly increased cytokines are usually different. There is often a difference between mild patients and severe patients with the same virus infection. Therefore, the kind of cytokine antagonists that should be used to control the inflammatory responses depends on individual situations. Many previous studies focused on IL-6, which may contribute to disease exacerbation, and some therapeutic approaches based on anti-IL-6 biologics have been proposed (22, 23) and validated (24). However, our analysis showed that besides IL-6, IP-10, IL-17, and IL-8 are the most common cytokines with the largest induction amplitudes (e.g., IL-8 may be the most significantly induced cytokine in SARS-CoV-2 infection) (25). Antagonists against IP-10, IL-17, or IL-8 have not attracted enough attention in clinical practice.

For the Th1-type infections, IFN-γ is the most important determinant of cytokine storm severity. Therefore, IFN-γ antagonists may be used as candidate drugs to control the inflammatory response. In most cases of Th1-type infections (IFN-γ FC/IL-4 FC > 1), IFN-γ is increased. There is one exception: although IFN-γ decreased after HBV infection, IL-4 decreased further (26); so, the ratio of IFN-γ FC/IL-4 FC was still greater than 1 (**Table S2**). In this case, IFN-γ antagonists should not be used. Given that max FC = (IFN-γ FC)^2^/IL-4 FC, enhancement to IL-4 may also prevent the cytokine storm. However, excessive IL-4 may suppress the inflammation too much and generate detrimental effects (15, 16). Over-inhibition to IFN-γ may also down-regulate immunity seriously, and therefore delay the clearance of the virus (15, 16). We should be very cautious when using either IFN-γ antagonists or IL-4.

For Th2-type infections (IFN-γ FC/IL-4 FC < 1), IFN-γ might be an ideal drug. IFN-γ has been proved to show some therapeutic effects to HIV patients (27). Our results suggest that IFN-γ treatment may also be extended to other Th2-type infections, such as the EV71 infection.

**Figure 4:**
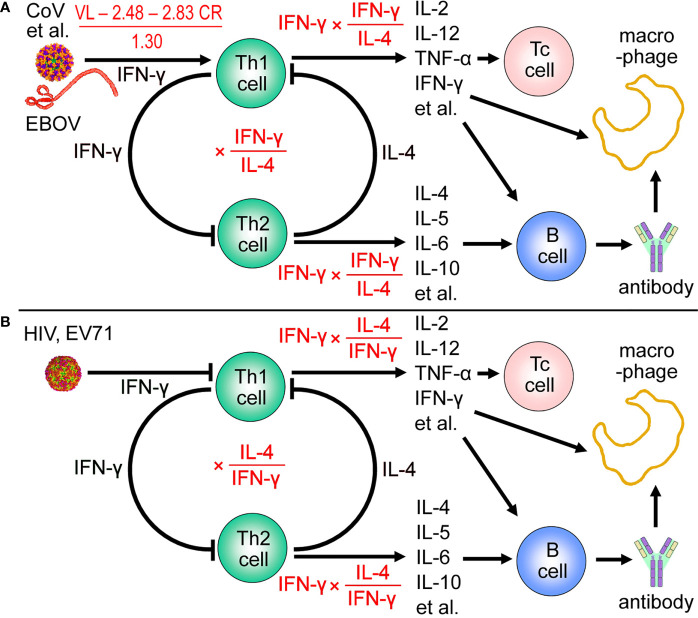
Viral load and clearance rate determine IFN-γ fold-change (FC) and amplitude of the maximum induced cytokine. **(A)** For T helper 1 (Th1) -type infections (except for hepatitis B virus), 1.30 × log_2_ (IFN-γ FC) = log_10_ (viral load; VL) − 2.48 − 2.83 × (clearance rate; CR). And the maximum induced cytokine (max) FC = IFN-γ FC × (IFN-γ FC/IL-4 FC). CoV, coronaviruses. CoV, coronaviruses. **(B)** For T helper 2 (Th2) -type infections, the maximum induced cytokine (max) FC = IFN-γ FC × (IL-4 FC/IFN-γ FC) = IL-4 FC. HCV, hepatitis C virus; EV71, enterovirus 71; Tc cell, cytotoxic T cell.

Our analysis suggests that viral replication and clearance rate are the decisive factors in inducing IFN-γ and other cytokines, thus determining severity of the cytokine storm (**Figure 4**). For example, the viral load of Ebola survivors was two orders of magnitude lower than Ebola fatalities (28), so a difference of 1.7 times (23.3 *vs* 40) in IFN-γ FC and a difference of 2.5 times (41.5 *vs* 102) in max FC were observed (**Table S2**) (11). 6.84 log_10_ 2009H1N1 virus particles could be cleared within 5 days (8, 9), whereas clearance of 7.07 log_10_ H7N9 virus requires 19.7 days (7, 29). Correspondingly, 1.3-fold and 9.3-fold inductions to IFN-γ were observed, respectively, and a difference of 5 times (2 *vs.* 10) in max FC was observed (**Table S2**) (10, 30). The viral load usually reaches a peak at 1–10 days post the infection, and the peak of cytokines often appears at the same time with the peak load or appears in 1–7 days post the peak load (**Tables S1–S4**). The best way to prevent a cytokine storm is to reduce the viral load or accelerate the virus clearance, which suggests that antiviral therapy should be started as early as possible.

A large number of studies have developed cytokine changes into boolean models, and then, converted the models to ordinary differential equations (ODEs) that helped to reveal the behaviour of the various cytokines (31–36). However, these reports mainly focused on dynamics of individual cytokines (31, 32, 34–36) or cytokine groups (such as pro-inflammatory cytokine group and anti-inflammatory cytokine group) (33). Interactions among IL-4, IFN-γ and cytokines with the largest induction amplitudes have not been modeled before. Nevertheless, our mathematical models are merely based on linear regression and quite simplified. Up till now, there are too few references for some infections (such as H3N2, H5N1, H7N9, Ebola, WNV and EV71) to construct a more sophisticated model. With more extensive research, more quantitative data about cytokine storms would be published, and then more precisely mathematical models may be established.

## Data Availability Statement

The original contributions presented in the study are included in the article/**Supplementary Material**. Further inquiries can be directed to the corresponding author.

## Author Contributions

SY had the idea for and designed the study and had full access to all data in the study and take responsibility for the integrity of the data and the accuracy of the data analysis. SY, and SCJ contributed to writing of the manuscript. All authors contributed to the literature search, data acquisition, data analysis, statistical analysis and reviewed and approved the final version.

## Funding

This work was supported by the Sichuan Province Youth Science and Technology Innovation Team (20CXTD0062) to SY and the Applied Basic Research Program of Sichuan Province (20YYJC4388) to Z-WZ.

## Conflict of Interest

S-CJ was employed by the Chengdu KangHong Pharmaceutical Group Comp. Ltd.

The remaining authors declare that the research was conducted in the absence of any commercial or financial relationships that could be construed as a potential conflict of interest.

## References

[1] Chan MC Cheung CY Chui WH Tsao SW Nicholls JM Chan YO. Proinflammatory Cytokine Responses Induced by Influenza A (H5n1) Viruses in Primary Human Alveolar and Bronchial Epithelial Cells. Respir Res (2005) 6:135. 10.1186/1465-9921-6-135 16283933 PMC1318487

[2] Cilloniz C Pantin-Jackwood MJ Ni C Goodman AG Peng X Proll SC. Lethal Dissemination of H5N1 Influenza Virus is Associated With Dysregulation of Inflammation and Lipoxin Signaling in a Mouse Model of Infection. J Virol (2010) 84:7613–24. 10.1128/JVI.00553-10 PMC2897611 20504916

[3] Fukuyama S Kawaoka Y. The Pathogenesis of Influenza Virus Infections: The Contributions of Virus and Host Factors. Curr Opin Immunol (2011) 23:481–6. 10.1016/j.coi.2011.07.016 PMC3163725 21840185

[4] Perrone LA Plowden JK Garcia-Sastre A Katz JM Tumpey TM. H5N1 and 1918 Pandemic Influenza Virus Infection Results in Early and Excessive Infiltration of Macrophages and Neutrophils in the Lungs of Mice. PloS Pathog (2008) 4:e1000115. 10.1371/journal.ppat.1000115 18670648 PMC2483250

[5] Wong CK Lam CW Wu AK Ip WK Lee NL Chan IH. Plasma Inflammatory Cytokines and Chemokines in Severe Acute Respiratory Syndrome. Clin Exp Immunol (2004) 136:95–103. 10.1111/j.1365-2249.2004.02415.x 15030519 PMC1808997

[6] Mahallawi WH Khabour OF Zhang Q Makhdoum HM Suliman BA. Mers-Cov Infection in Humans is Associated With a Pro-Inflammatory Th1 and Th17 Cytokine Profile. Cytokine (2018) 104:8–13. 10.1016/j.cyto.2018.01.025 29414327 PMC7129230

[7] Zhang Y Gao H Liang W Tang L Yang Y Wu X. Efficacy of Oseltamivir-Peramivir Combination Therapy Compared to Oseltamivir Monotherapy for Influenza A (H7n9) Infection: A Retrospective Study. BMC Infect Dis (2016) 16:76. 10.1186/s12879-016-1383-8 26864456 PMC4748590

[8] Duchamp MB Casalegno JS Gillet Y Frobert E Bernard E Escuret V. Pandemic A(H1N1)2009 Influenza Virus Detection by Real Time RT-PCR: Is Viral Quantification Useful? Clin Microbiol Infect (2010) 16:317–21. 10.1111/j.1469-0691.2010.03169.x 20121827

[9] Yu H Liao Q Yuan Y Zhou L Xiang N Huai Y. Effectiveness of Oseltamivir on Disease Progression and Viral Rna Shedding in Patients With Mild Pandemic 2009 Influenza A H1n1: Opportunistic Retrospective Study of Medical Charts in China. BMJ (2010) 341:c4779. 10.1136/bmj.c4779 20876641 PMC2947622

[10] Bermejo-Martin JF Ortiz de Lejarazu R Pumarola T Rello J Almansa R Ramírez P. Th1 and Th17 Hypercytokinemia as Early Host Response Signature in Severe Pandemic Influenza. Crit Care (2009) 13:R201. 10.1186/cc8208 20003352 PMC2811892

[11] Kerber R Krumkamp R Korva M Rieger T Wurr S Duraffour S. Kinetics of Soluble Mediators of the Host Response in Ebola Virus Disease. J Infect Dis (2018) 218(S5):S496–503. 10.1093/infdis/jiy429 PMC6249596 30101349

[12] Yong X Liu Z Jiang L Tao R Liu W Zhang L. Dynamic Changes of Th1/Th2/Th17 Cytokines and Human Beta Defensin 2 in HIV-infected Patients With Oral Candidiasis During the First Year of Highly Active Anti-Retroviral Therapy. Arch Oral Biol (2018) 92:62–7. 10.1016/j.archoralbio.2018.05.003 29753928

[13] Zhang Y Liu H Wang L Yang F Hu Y Ren X. Comparative Study of the Cytokine/Chemokine Response in Children With Differing Disease Severity in Enterovirus 71-Induced Hand, Foot, and Mouth Disease. PloS One (2013) 8:e67430. 10.1371/journal.pone.0067430 23840697 PMC3696071

[14] Zhang SY Xu MY Xu HM Li XJ Ding SJ Wang XJ. Immunologic Characterization of Cytokine Responses to Enterovirus 71 and Coxsackievirus A16 Infection in Children. Medicine (2015) 94:e1137. 10.1097/MD.0000000000001137 26166120 PMC4504596

[15] Paludan SR. Interleukin-4 and Interferon-Gamma: The Quintessence of a Mutual Antagonistic Relationship. Scand J Immunol (1998) 48:459–68. 10.1046/j.1365-3083.1998.00435.x 9822252

[16] Infante-Duart C Kamradt T. Thl/Th2 Balance in Infection. Springer Semin Immunopathol (1999) 21:317–38. 10.1007/BF00812260 10666776

[17] Sissoko D Duraffour S Kerber R Kolie JS Beavogui AH Camara AM. Persistence and Clearance of Ebola Virus RNA From Seminal Fluid of Ebola Virus Disease Survivors: A Longitudinal Analysis and Modelling Study. Lancet Glob Health (2017) 5:e80–8. 10.1016/S2214-109X(16)30243-1 27955791

[18] Tricou V Minh NN Farrar J Tran HT Simmons CP. Kinetics of Viremia and NS1 Antigenemia are Shaped by Immune Status and Virus Serotype in Adults With Dengue. PloS Negl Trop Dis (2011) 5:e1309. 10.1371/journal.pntd.0001309 21909448 PMC3167785

[19] Hofer H Watkins-Riedel T Janata O Penner E Holzmann H Steindl-Munda P. Spontaneous Viral Clearance in Patients With Acute Hepatitis C can be Predicted by Repeated Measurements of Serum Viral Load. Hepatology (2003) 37:60–4. 10.1053/jhep.2003.50019 12500189

[20] Thomson EC Fleming VM Main J Klenerman P Weber J Eliahoo J. Predicting Spontaneous Clearance of Acute Hepatitis C Virus in a Large Cohort of HIV-1-infected Men. Gut (2011) 60:837–45. 10.1136/gut.2010.217166 PMC3095479 21139063

[21] To KK Tsang OT Leung WS Tam AR Wu TC Lung DC. Temporal Profiles of Viral Load in Posterior Oropharyngeal Saliva Samples and Serum Antibody Responses During Infection by SARS-CoV-2: An Observational Cohort Study. Lancet Infect Dis (2020) 20:565–74. 10.1016/S1473-3099(20)30196-1 PMC7158907 32213337

[22] Copaescu A Smibert O Gibson A Phillips EJ Trubiano JA. The Role of IL-6 and Other Mediators in the Cytokine Storm Associated With SARS-CoV-2 Infection. J Allergy Clin Immunol (2020) 146:518–34.e1. 10.1016/j.jaci.2020.07.001 PMC7471766 32896310

[23] Gubernatorova EO Gorshkova EA Polinova AI Drutskaya MS. Il-6: Relevance for Immunopathology of SARS-Cov-2. Cytokine Growth Factor Rev (2020) 53:13–24. 10.1016/j.cytogfr.2020.05.009 32475759 PMC7237916

[24] Masiá M Fernández-González M Padilla S Ortega P García JA Agulló V. Impact of Interleukin-6 Blockade With Tocilizumab on SARS-CoV-2 Viral Kinetics and Antibody Responses in Patients With Covid-19: A Prospective Cohort Study. EBioMedicine (2020) 60:102999. 10.1016/j.ebiom.2020.102999 32950003 PMC7492814

[25] Huang C Wang Y Li X Ren L Zhao J Hu Y. Clinical Features of Patients Infected With 2019 Novel Coronavirus in Wuhan, China. Lancet (2020) 395:497–506. 10.1016/S0140-6736(20)30183-5 31986264 PMC7159299

[26] Shata MTM Abdel-Hameed EA Rouster SD Yu L Liang M Song E. HBV and HIV/HBV Infected Patients Have Distinct Immune Exhaustion and Apoptotic Serum Biomarker Profiles. Pathog Immun (2019) 4:39–65. 10.20411/pai.v4i1.267 30815625 PMC6388707

[27] Poli G Biswas P Fauci AS. Interferons in the Pathogenesis and Treatment of Human Immunodeficiency Virus Infection. Antiviral Res (1994) 24:221–33. 10.1016/0166-3542(94)90069-8 7526793

[28] Towner JS Rollin PE Bausch DG Sanchez A Crary SM Vincent M. Rapid Diagnosis of Ebola Hemorrhagic Fever by Reverse transcription-PCR in an Outbreak Setting and Assessment of Patient Viral Load as a Predictor of Outcome. J Virol (2004) 78:4330–41. 10.1128/jvi.78.8.4330-4341.2004 PMC374287 15047846

[29] Zhu Z Liu Y Xu L Guan W Zhang X Qi T. Extra-Pulmonary Viral Shedding in H7N9 Avian Influenza Patients. J Clin Virol (2015) 69:30–2. 10.1016/j.jcv.2015.05.013 26209373

[30] Yang Y Wong G Yang L Tan S Li J Bai B. Comparison Between Human Infections Caused by Highly and Low Pathogenic H7N9 Avian Influenza Viruses in Wave Five: Clinical and Virological Findings. J Infect (2019) 78:241–8. 10.1016/j.jinf.2019.01.005 30664912

[31] Seymour RM Henderson B. Pro-Inflammatory–Anti-Inflammatory Cytokine Dynamics Mediated by Cytokine-Receptor Dynamics in Monocytes. IMA J Math Appl Med Biol (2001) 18:159–92. 10.1093/imammb/18.2.159 11453467

[32] Yiu HH Graham AL Stengel RF. Dynamics of a Cytokine Storm. PloS One (2012) 7:e45027. 10.1371/journal.pone.0045027 23049677 PMC3462188

[33] Jarrett AM Cogan NG Shirtliff ME. Modelling the Interaction Between the Host Immune Response, Bacterial Dynamics and Inflammatory Damage in Comparison With Immunomodulation and Vaccination Experiments. Math Med Biol (2015) 32:285–306. 10.1093/imammb/dqu008 24814512 PMC4416065

[34] Waito M Walsh SR Rasiuk A Bridle BW Willms AR. A Mathematical Model of Cytokine Dynamics During a Cytokine Storm. In: Bélair J Frigaard I Kunze H Makarov R Melnik R Spiteri R, editors. Mathematical and Computational Approaches in Advancing Modern Science and Engineering. Cham, Switzerland: Springer, Cham (2016). p. 331–9. 10.1007/978-3-319-30379-6_31

[35] Waters RS Perry JSA Han S Bielekova B Gedeon T. The Effects of Interleukin-2 on Immune Response Regulation. Math Med Biol (2018) 35:79–119. 10.1093/imammb/dqw021 28339682 PMC5576036

[36] Zhang W Jang S Jonsson CB Allen LJS. Models of Cytokine Dynamics in the Inflammatory Response of Viral Zoonotic Infectious Diseases. Math Med Biol (2019) 36:269–95. 10.1093/imammb/dqy009 PMC7108568 29961899

