# Supplementary material for: Quantification of Cytokine Storms During Virus Infections
Source: Front Immunol. 2021 May 17;12:659419. doi: 10.3389/fimmu.2021.659419 (PMC8165266; doi:10.3389/fimmu.2021.659419)
Supplement: Supplementary file 1 [file DataSheet_1.pdf]

Table S1. Cytokine fold-changes after different virus infections.

Virus	Cytokines	Adjustments and error bars (n)	Reference
SARS	IL-12, IL-10, TNF-α, IL-8, IFN-γ, MCP-1, IL-6, IP-10, IL-1b, MIG, RANTES	Bars show interquartile ranges (n=20).	Wong et al., Clin Exp Immunol 2004; 136: 95–103
SARS	TNF-α, IL-1a, IL-6, IFN-γ, IL-8, TGF-β, IL-4, IL-10	Bars show SEM (n=30).	Zhang et al., Infect Immun 2004; 72: 4410–4415
SARS	IP-10, IL-6, IL-8, IFN-γ	MCP-1 and MIP-1a are excluded due to their extremely low levels in the control sample. Bars show SD (n=23).	Jiang et al., Am J Respir Crit Care Med 2005; 171: 850–857
SARS	IL-6, IL-8, IL-13, IL-16, IL-18, TNF-α, TGF-β1	Bars show SD (n=61).	Beijing Group of National Research Project for SARS. Chin Med J (Engl) 2003; 116: 1283–1287
MERS	IL-17, IL-2, IL-4, IL-5, IL-13, TGF	IFN-α2, IFN-γ, IL-12, IL-15, IL-10, IL-17, TNF-α are excluded due to their extremely low levels in the control sample. Bars show SEM (n=7).	Mahallawi et al., Cytokine 2018; 104: 8–13
MERS	IFN-α, MCP-1, TNF-α, MIP-1a, RANTES, IL-6, IL-12, IL-8, IFN-β, IFN-γ, IP-10	In human monocyte-derived macrophages. Bars show SEM (n≥3).	Zhou et al., J Infect Dis 2014; 209: 1331–1342
SARS-CoV-2	IL-10, MIP-1b, IL-4, IL-17, IL-9, TNF-α, G-CSF, MIP-1a, MCP-1, IL-6, IL-1b, IL-1Ra, IL-12, PDGF-bb, IL-7, IL-2, IFN-γ, VEGF, IL-8, IL-5, IL-13, IL-15, Eotaxin, FGF, GM-CSF, IP-10, RANTES	IL-6 in health control was too low and referenced to 2 pg/ml (Wong et al. J Infect Dis 2018; 217: 245–256). Bars show interquartile ranges. ICU patients (n=13); non-ICU patients (n=28).	Huang et al., Lancet 2020; 395: 497–506
SARS-CoV-2	IFN-γ, IL-5, IL-8, Il-9, IL-17, TGF-β,	Bars show SEM. Mild (n=33); severe (n=30).	Ghazavi et al., Cytokine 2020; doi:10.1016/j.cyto.2020.155323
SARS-CoV-2	IL-6, MCP-3, PTX3, GDF-2	Bars show SEM. Mild (n=5); severe (n=9).	Sims et al., J Allergy Clin Immunol 2020; doi:10.1016/j.jaci.2020.08.031
SARS-CoV-2	IFN-γ, TNF-α, IL-2,	Bars show SD (n=102).	Han et al., Emerg Microbes

	IL-4, IL-6, IL-10		Infect 2020; 9: 1123–1130
SARS-CoV-2	IL-1β, IL-1Ra, IL-6, IL-13, IL-18, MCP-3, MIG, M-CSF, G-CSF, IL-2, IL-7, IL-9, IL-10, IL-12, IL-13, IL-15, IL-3, MIP-1α, MIP-1β, IP-10, IFN-γ	Bars show SEM. moderate (n=14); severe (n=25).	Yang et al., J Allergy Clin Immunol 2020; 146: 119–127
2009H1N1	IL-15, IL-13, IFN-γ, IL-8, IL-12, IL-6, TNF-α, IL-17	IL-9 is excluded due to their extremely low levels in the control sample. Bars show interquartile ranges. Outpatients (n=31); non critical patients (n=14); critical patients (n=21).	Bermejo-Martin et al., Crit Care 2009; 13: R201
2009H1N1	PDGF-bb, IFN-γ, IL-6, IP-10, VEGF, PAI-1, TNF-α, MCP-1, IL-8, FGF	Bars show SEM. ARDS (n=15); ARDS + acute kidney injury (n=17).	Bautista et al., Exp Mol Pathol 2013; 94: 486–492
2009H1N1	IL-6, IL-8, IL-9, IL-12, IL-15, IFN-γ	IL-17, IP-10 and TNF-α are excluded due to their extremely low levels in the control sample. Bars show interquartile ranges. Mild disease group (n=11); ARDS group (n=21); ARDS bacterial sepsis group (n=20).	Hagau et al., Crit Care 2010; 14: R203
2009H1N1	IL-1Ra, IL-2, IL-4, IL-5, IL-6, IL-7, IL-8, IL-9, IL-10, IL-12, IL-13, IL-15, IL-17, Eotaxin, IFN-γ, IP-10, MCP-1, RANTES, TNF-α	Bars show SD (n=58).	Yang et al., Clin Respir J 2014; 8: 185–191
2009H1N1	IL-4	IL-4 in health control was referenced to 33 pg/ml (Martinez-Ocaña et al. J Clin Virol 2013; 58: 108–113). Bars show SEM. With pneumonia (n=27); without pneumonia (n=20).	Matsumoto et al., Microbiol Immunol 2012; 56: 651–655 Martinez-Ocaña et al. J Clin Virol 2013; 58: 108–113
H3N2	IL-4, IL-5, G-CSF, MCP-1, IFN-γ, TNF-α, VEGF, GRO, PDGF-bb, EGF, IL-8, SCD40L, IL-3,	Among 40 cytokines, 20 cytokines associated with severe acute respiratory illness were selected plus IFN-γ. Bars represent the 95%	Wong et al. J Infect Dis 2018; 217: 245–256

	TNF-β, IL-13, GM-CSF, IFN-α2, fkn, PDGF-aa, IL-12p40, IL-12p70	confidence-interval of the calculated mean. Influenza-like illness (mild; n=27); SARI (n=27).	
H3N2	IL-1b, IL-6, IL-17, IL-22, IL-23	Bars show SEM (n=10).	Antalis et al., J Med Virol 2019; 91: 963–971
H3N2	IL-1Ra, IL-2, IL-4, IL-5, IL-6, IL-7, IL-8, IL-9, IL-10, IL-12, IL-13, IL-15, IL-17, Eotaxin, IFN-γ, IP-10, MCP-1, RANTES, TNF-α	Bars show SD (n=30).	Yang et al., Clin Respir J 2014; 8: 185–191
H7N9	MCP-1, MIF, IP-10, IFN-γ, SCF, IL-8, MIG, IL-16	HGF is excluded due to their extremely low levels in the control sample. Bars show SEM. Low pathogenic virus (n=7); high pathogenic virus (n=5).	Yang et al., J Infect 2019; 78: 241–248
H7N9	IL-1b, IL-2, IL-4, IL-6, IL-9, IL-10, IL-17, TNF-α, IFN-γ, RANTES, Eotaxin, MIP-1, MCP-1	Bars show SD (n=11).	Han et al., Microbes Infect 2014; 16: 672–677
H7N9	MIP-1b	MCP-1, IL-6, IP-10, IFN-α, IFN-γ, IL-8 and MIG are excluded due to their extremely low levels in the control sample. Bars show SEM (n=7).	Zhou et al., Nature 2013; 499: 500–503
H7N9	IL-4	IL-6, IL-8, IL-10 and MIP1a are excluded due to their extremely low levels in the control sample. Bars show SEM. Low pathogenic virus (n=9); high pathogenic virus (n=6).	Mao et al., J Clin Virol 2015; 63: 18–24
H5N1	MIP-1b, IFN-γ, IP-10, IFN-α, MCP-1, MIG	IL-6 and IL-8 are excluded due to their extremely low levels in the control sample. Bars show SEM (n=11).	Zhou et al., Nature 2013; 499: 500–503
H5N1	IL-4, IFN-γ	No report of IL-4 in humans could be found. Data in mice were used instead. Bars show SEM (n=6).	Tao et al., Can J Microbiol 2012; 58: 391–401
Ebola	IL-2, IL-6, IL-10, IFN-α, IFN-γ, TNF-α	IL-1b and IL-12b are excluded due to their extremely low levels	Villinger et al., J Infect Dis 1999; 179 (Suppl 1):

		in the control sample. Bars show SD. Fatal cases (n=7).	S188–S191
Ebola	IL-1Ra, sTNF-RI, sTNF-RII, SIL-6R, MIP-1a, MIP-1b,	IL-1b, TNF-α, IL-6 and IL-10 are excluded due to their extremely low levels in the control sample. Bars show SD. Fatalities (n=12); survivors (n=5).	Baize et al., Clin Exp Immunol 2002; 128: 163–168
Ebola	IFN-α, TNF-α, IL-6, IL-8, IL-18, IL-1a	Error bars define the 10th and 90th percentiles. Fatal cases (n=44); survivors (n=58).	Reynard et al., JCI Insight 2019; 4: e125106
Ebola	IL-1a, IL-1b, TNF-α, IL-6, IL-10, IFN-γ, IFN-α, MCP-1	Bars show SEM (n=24).	Gupta et al., Virology 2012; 423: 119–124
Ebola	IL-1b, IL-2, IL-4, IL-6, IFN-α, IFN-γ, MIP-1a, MIP-1b, GM-CSF, MCP-2, IL-29, TNF-α, EGF, G-CSF, Fractalkine, GRO, sCD40L, sTNF-RI, sTNF-RII IL-1, IL-5, IL-8, IP-10, MCP-1, sTNF-RI, sTNF-RII, VEGF, RANTES, IFN-β	M-CSF, IL-10, MCP-3, IL-12, IL-1Ra and sIL-1RII are excluded due to their extremely low levels in the control sample. Bars show interquartile ranges. Fatalities (n=202); survivors (n=186).	Kerber et al., J Infect Dis 2018; 218(S5): S496–S503
HIV	IL-4, IL-10, TGF-β, IL-6, IFN-γ, TNF-α	Bars show SD. Before HAART (n=50); 1 year after HAART (n=50).	Osuji et al., J Biomed Sci 2018; 25: 88
HIV	IL-4, IL-17, IFN-γ, HBD-2	Bars show SD. Before HAART (n=25); 1 year after HAART (n=25).	Yong et al., Arch Oral Biol 2018; 92: 62–67
HIV	IL-2, IFN-γ, TNF-α, IL-4, IL-10, IL-6	Bars show interquartile ranges (n=22).	Bal et al., Cytokine 2005; 30: 275–281
HIV	TNF-α, IL-4, IL-10, IFN-γ	Bars show SD (n=32).	Altfeld et al., J Acquir Immune Defic Syndr 2000; 23: 287–294
HIV	IP-10, MIG, MIP-1a, MIP-1b, IFN-γ, TNF-α, RANTES, GM-CSF, IL-4, IL-10, IL-6, IL-18,	No error bars (n=5).	Teruya-Feldstein et al., Mod Pathol 2001; 14: 929–936
DENV	IL-1b, IL-2, IL-4, IL-5, IL-6, IL-10, IL-13, IFN-γ, TNF-α,	Bars show SD. Dengue fever (n=40); Dengue hemorrhagic fever (n=20).	Wang et al., Int J Infect Dis 2019; 88: 88–99

	GM-CSF, MCP-1, MIP-1b		
DENV	IFN-γ, TNF-α, MIF	IL-15 and IL-10 are excluded due to their extremely low levels in the control sample. Bars show interquartile ranges (n=30).	Sánchez-Arcila et al. Intervirology 2020; doi:10.1159/000510223
DENV	IL-1b, IL-12	TNF-α is excluded due to their extremely low level in the control sample. Bars show interquartile ranges. Mild (n=148); severe (n=7).	Tuyen et al., Viral Immunol 2020; 33: 514–520
DENV	TNF-α, IL-8, IL-15, GM-CSF, MCP-1, IL-10, IFN-γ, MIP-1b, IL-6	Bars show SEM (n=47). Mild (n=35); severe (n=12).	Patro et al., Viruses 2019; 11: 34
DENV	IL-8, IL-18, MIP-1a, RANTES, MIP-1b	IL-10, IFN-γ, Bars show SD. Primary infections (n=234); secondary infections (n=63)	Huang et al., Dis Markers 2018; 2018: 8403937
DENV	IFN-γ, TNF-α,, IL-12, IL-6, IL-8	Bars show SD. Dengue fever (n=101); Dengue hemorrhagic fever (n=70).	Cruz Hernández et al., Mem Inst Oswaldo Cruz 2016; 111: 161–167
DENV	IFN-γ, TNF-α, IL-6, IL-8	Bars show SEM. Dengue fever (n=159); Dengue hemorrhagic fever (n=62).	Priyadarshini et al., PLoS One 2010; 5: e8709
ZIKV	IL-13, IL-15, TNF-α, IL-1b, IL-2, IL-4, GM-CSF, IL-12, G-CSF, IFN-α, IL-1Ra, M-CSF, IFN-γ, IL-5, IL-10, IL-6, IL-17, sCD40L	Bars show interquartile ranges (n=44).	Fares-Gusmao et al., Sci Rep 2019; 9: 17172
ZIKV	IFN-γ, TNF-α, MIF	IL-15 and IL-10 are excluded due to their extremely low levels in the control sample. Bars show interquartile ranges (n=22).	Sánchez-Arcila et al. Intervirology 2020; doi:10.1159/000510223
ZIKV	IL-9, IL-17, IL-1Ra	Bars represent the 95% confidence-interval of the calculated mean (n=23).	Zuñiga et al., Int J Infect Dis 2020; 94: 4–11
ZIKV	IL-6, IL-12, TNF-α	IFN-α, IFN-β and IFN-γare excluded due to their extremely low levels in the control sample. Bars show SEM (n=30).	da Silva et al., Med Microbiol Immunol 2019; 208: 703–714
ZIKV	IL-1b, IL-6, TNF-α, IL-12, IFN-γ, IL-17,	Bars show interquartile ranges (females, n=29).	Naveca et al., Mem Inst Oswaldo Cruz 2018; 113:

	IL-1Ra, IL-4, IL-5, IL-9, IL-10, IL-13, FGF, PFGF, VEGF, G-CSF, GM-CSF		e170542
WNV	IL-13, IL-15, TNF-α, IL-1b, IL-2, IL-4, GM-CSF, IL-12, G-CSF, IFN-α, IL-1Ra, M-CSF, IFN-γ, IL-5, IL-10, IL-6, IL-17, sCD40L	Bars show interquartile ranges (n=52).	Fares-Gusmao et al., Sci Rep 2019; 9: 17172
HCV (untreated acute infection)	IL-4, TNF-α, IL-6, IL-2, IL-10, IL-17	IFN-γ is excluded due to its extremely low level in the control sample. Bars show SEM. G1 genotype (n=47); G2 and G3 genotype (n=22).	Tarragô et al., Hum Immunol 2014; 75: 1075–1083
HCV (chronic)	IFN-γ, IL-18, IL-4, IL-10	No report of IFN-γ in untreated patients could be found. Data in chronic (treated) HCV infection were used instead. Bars show SD (n=20).	El-Kady et al., Scand J Immunol 2005; 61: 87–91
HCV	IL-2, IL-4, IL-10, IFN-γ	Bars show SEM (n=63).	Sofian et al., Hepat Mon 2012; 12: e6156
HCV	IL-2, IL-4, IL-10, IFN-γ	Bars show SEM (n=18).	Fan et al., Mediators Inflamm 1998;7: 295–297
HCV	IL-2, IL-4, IL-10, IFN-γ	Bars show SD. Occult (n=35); chronic (n=50);.	Mousa et al., Int J Immunopathol Pharmacol 2014; 27: 87–96
HCV	TNF-α, IL-1b, IL-6, IFN-γ, IL-2, IL-4, IL-10, IL-12	Bars show SEM (n=30).	Ríos-Olivares et al., Drug Alcohol Depend 2006; 85: 236–243
HCV	IL-1b, IL-1Ra, IL-2, IL-4, IL-5, IL-6, IL-7, IL-8, IL-9, IL-10, IL-12, IL-13, IL-15, IL-17, Eotaxin, FGF, G-CSF, GM-CSF, IFN-γ, IP-10, MCP-1, MIP-1a, MIP-1b, RANTES, TNF-α, VEGF, PDGF-bb	Bars show SD (n=37).	Zhang et al., Antiviral Res 2011; 92: 247–254
HCV	IFN-γ, IL-17, IL-21, IL-22, TGF-β	Bars show SD (n=28).	Kong et al., Virol J 2015; 12: 100
HCV	IFN-γ, IL-1b, IL-10,	Bars show interquartile ranges	Saraiva et al., Mediators

	IL-1Ra, TGF-β, FGF-β, PDGF-bb, VEGF	(n=88).	Inflamm 2018; 2018: 8578051
HBV	IL-4, IL-2, GM-CSF, IL-7, IL-5, IL-13, IFN-γ, IL-1b, IL-12p70, TNF-α, IL-6, IL-8, IL-10, TGF-β1, sPD-1, sPD-L1	Bars show SD (n=30).	Shata et al., Pathog Immun. 2019; 4: 39–65
HBV	IL-1b, IL-2, TNF-α	IL-6, IL-8 and IL-10 are excluded due to their extremely low levels in the control sample. Bars show interquartile ranges (n=18).	Wang et al., Hepat Mon 2014; 14: e19370
HBV	IL-2, IL-4, IL-6, IL-7, IL-9, IL-10, IL-12, IL-15, IL-21, IL-23, IL-28, IL-29	Bars show SD (n=10).	He et al., J Clin Immunol 2013; 33: 1240–1249
HBV	IFN-γ, IL-4, TGF-β1, TNF-α	Bars show SD (n=30).	Akpolat et al., World J Gastroenterol 2005; 11: 3260–3263
EV71	IFN-γ, IL-5, IL-17, IL-13, IL-23, IL-10, IL-6, IL-2, IL-1b, IL-4, TNF-α, IL-22	Bars show 95% confidence-interval of the calculated mean. Encephalitis (n=40); without encephalitis (n=52).	Zhang et al., Medicine 2015; 94: e1137
EV71	IL-6, IL-10, IL-13	Bars show 95% confidence intervals (Stage IV, n=22).	Chen et al., Int J Clin Exp Med 2014; 7: 2718–2723
EV71	G-CSF, MCP-1, MIP-1b, GM-CSF, IL-33, IFN-α, MIP-1a,	IL-2, IL-23, IP-10, IL-6 and IL-8 are excluded due to their extremely low levels in the control sample. Bars show SEM. Severe (n=23); mild (n=19).	Zhang et al., PLoS One 2013; 8: e67430
EV71	IL-6, IL-23, TNF-α	IL-22 and IL-17A are excluded due to their extremely low levels in the control sample. Bars show SEM. Severe (n=11); mild (n=18).	Cui et al., Oncotarget 2017; 8: 29370–29382
EV71	IL-2, IL-16, IFN-γ, GM-CSF, IL-4, IL-6, IL-10, IL-1b, TNF-α	Bars show SD (n=16).	Wang et al., Glob Pediatr Health 2016; 3: 2333794X16643723

HAART, highly active antiretroviral therapy; SD, standard deviation; SEM, standard error of mean; ARDS, acute respiratory distress syndrome; ICU, intensive care unit.

Table S2. Fold-changes of IFN-γ, IL-4 and the two maximum induced cytokines after different virus infections.

Virus	IFN-γ	IL-4	IFN-γ/ IL-4	two maximum induced cytokines
SARS	6	1.1	5.45	33 (IL-1b); 14 (IP-10)
MERS	6	1.3	4.62	27.6 (IL-17); 9 (IP-10)
Non-ICU of SARS-CoV-2	7	1.4	5.00	30 (IL-8); 8.3 (VEGF)
ICU of SARS-CoV-2	8	1.5	5.33	40 (IL-8); 13.8 (MCP-3)
2009H1N1 mild	1.3	1	1.30	2 (IL-6); 1.8 (IL-12)
2009H1N1 severe	2.5	0.91	2.75	6 (MCP-1); 5.6 (FGF)
2009H1N1 critical	3	0.68	4.41	12.7 (MCP-1); 12 (IL-6)
H3N2 mild	1.07	0.96	1.12	1.31 (IL-8); 1.26 (sCD40L)
H3N2 severe	1.14	0.88	1.30	1.71 (IL-22); 1.55 (IL-6)
H7N9 mild	8	4	2.00	8 (IFN-γ); 6 (MIF)
H7N9 severe	9.3	10	0.93	10 (IL-4); 9.7 (IP-10)
H5N1	6	3	2.00	11 (MIG); 10 (MCP-1)
Ebola survivors	23.3	14.7	1.59	41.5 (IP-10); 27.4 (IL-8)
Ebola fatalities	40	14.7	2.72	102 (IL-8); 55 (IP-10)
Dengue fever	8.9	5.7	1.56	11.1 (IL-6); 8.9 (IFN-γ)
Dengue hemorrhagic fever	22.2	14.3	1.55	33.3 (IL-6); 22.2 (IFN-γ)
ZIKV	4.5	1.3	3.46	12.6 (IL-17); 10 (IL-5)
WNV	20	10	2.00	39.8 (IL-17); 20 (IFN-γ)
HIV before HAART	0.16	7.2	0.0222	7.2 (IL-4); 6.8 (IL-10)
HIV 1 yr after HAART	0.54	2.5	0.216	2.5 (IL-4); 2.5 (HBD-2)
HBV	0.89	0.35	2.54	2.5 (IL-29); 2.21 (sPD-1)
HCV (G1)	4.3	0.33	13.0	50 (IL-17); 12 (GM-CSF)
HCV (G2 & G3)	4.3	0.67	6.42	26 (IL-17); 12 (GM-CSF)
EV71 with encephalitis	0.5	10.4	0.0481	11.5 (IL-13); 10.4 (IL-4)
EV71 without encephalitis	0.3	4	0.0750	5.5 (IL-22); 5.5 (MIP-1a)

HAART, highly active antiretroviral therapy; ICU, intensive care unit.

Table S3. IFN-γ fold-change, viral load and duration of virus of different infections.

Virus	IFN-γ fold-change	Viral load (reference)	Duration of virus (days)
SARS	6	5.8 $\log_{10}$ (n=142; Hung et al., Emerg Infect Dis 2004; 10: 1550–1557)<br>1.9 × 10^7 (n=20; Peiris et al., Lancet 2003; 361: 1767–1772)	21 in sputa (n=56; Liu et al., Emerg Infect Dis 2004; 10: 1841–1843)
MERS	6	5 × 10^6 (n=37; Corman et al., Clin Infect Dis 2016; 62: 477–483)<br>7.21 $\log_{10}$ (severe, n=9); 5.54 $\log_{10}$ (mild, n=8; Oh et al., N Engl J Med 2016; 375: 1303–1305)	19 (n=30; Hong et al., Thorax 2018; 73: 286–289)
SARS-CoV-2	7 (mild);<br>8 (severe)	1.18 × 10^6 (n=9; Wölfel et al., Nature 2020; 581: 465–469)<br>6.4 $\log_{10}$ (n=246; Yazdanpanah et al., 2020; doi:10.1002/jmv.26601)<br>5.2 $\log_{10}$ (n=30; To et al., Lancet Infect Dis. 2020; 20: 565–574)	12-33 days as indicated in 12 references. However most of them did not discriminate the severity. Zheng et al. (BMJ 2020; 369: m1443) showed 14 days and 21 days for mild disease (n=22) and severe disease (n=74) respectively.
2009H1N1	1.3 (mild);<br>2.5 (severe);<br>3 (critical)	5.97 $\log_{10}$ (mild; n=29); 5.68 $\log_{10}$ (severe; n=10); 5.45 $\log_{10}$ (critical; n=18; To et al., Clin Infect Dis 2010; 50: 850–859)<br>6.84 $\log_{10}$ (n=209; Duchamp et al., Clin Microbiol Infect 2010; 16: 317–321)<br>4.6 $\log_{10}$ (n=144; Panning et al., Euro Surveill 2009; 14: 19329)<br>6.4 $\log_{10}$ (n=18; Wu et al., Influenza Other Respir Viruses 2012; 6: 367–373)	5 (n=72; Na et al., J Med Virol 2011; 83: 5–9)<br>12 (n=10; Alves et al., Mem Inst Oswaldo Cruz 2020; 115: e200009)<br>4.2 (n=35; Suryaprasad et al., Clin Infect Dis 2011; 52(S1): S109–S115)<br>5 (n=1289; Yu et al., BMJ 2010; 341: c4779)<br>6 (n=426; Cao et al., N Engl J Med 2009; 361: 2507–2517)
H3N2	1.07 (mild);<br>1.14 (severe)	4.51 $\log_{10}$ (mild, n=16); 5.96 $\log_{10}$ (severe, n=22; Lee et al., J Infect Dis 2009; 200: 492–500)<br>6.94 $\log_{10}$ (n=267; Granados et al., J Clin Virol 2017; 86: 14–19)	3.4 (n=36; Loeb et al., J Infect Dis 2012; 206: 1078–1084)<br>5.14 (n=228; Carrat et al., Am J Epidemiol 2008; 167: 775–785)
H7N9	8.0 (mild);<br>9.3 (severe)	3.0 $\log_{10}$ (mild, n=12); 5.3 $\log_{10}$ (severe, n=6; Shen et al., Clin Microbiol Infect 2014; 20: O493–O500)<br>6.34-6.53 $\log_{10}$ (all patients, n=82); 6.58-7.07 $\log_{10}$ (ARDS	19.7 (severe, n=18; Zhu et al., J Clin Virol 2015: 69: 30–32)

		patients, n=72; Zhang et al., BMC Infect Dis 2016; 16: 76)	
H5N1	6	5.9 $\log_{10}$ (non-fatal; n=5); 7.5 $\log_{10}$ (fatal, n=13; de Jong et al., Nat Med 2006; 12: 1203–1207)	27 (n=5; de Jong et al., Nat Med 2006; 12: 1203–1207)
Ebola	23.3 (survivors);<br>40 (fatalities)	4.3×10^7 (non-fatal, n=18); 3.4×10^9 (fatal, n=27; Towner et al., J Virol 2004; 78: 4330–4341)	158 (n=26; Sissoko et al., Lancet Glob Health 2017; 5: e80–e88)
HIV	0.16 (before HAART)<br>0.54 (after HAART)	4.0-7.0 $\log_{10}$ as indicated in 127 references. One example with the largest n: 4.42 $\log_{10}$ (n=6438; Mark et al., AIDS Res Treat 2019; 2019: 9271450)	31 (n=50; Robb et al., N Engl J Med. 2016; 374: 2120–2130)
DENV	8.9 (Dengue fever);<br>22.2 (Dengue hemorrhagic fever)	1.69-5.46 × 10^8 (n=40; Nunes et al., Viruses 2018; 10: 326)<br>2.05-7.00 × 10^5 (n=101; Mukherjee et al., Eur J Clin Microbiol Infect Dis. 2017; 36: 2273–2279)<br>7.7 $\log_{10}$ (Dengue fever, n=54); 9.0 $\log_{10}$ (Dengue hemorrhagic fever, n=49; Wang et al., Clin Infect Dis 2006; 43: 1023–1030)	6 (n=248; Tricou et al., PLoS Negl Trop Dis 2010; 4: e785)<br>6 (n=239); Although the time to resolution of viremia in primary infections was about 6 days, only 20% of these patients had cleared NS1 viral protein when discharged from hospital. Time to resolution of NS1 antigenemia could be inferred to be about 12 days (Tricou et al., PLoS Negl Trop Dis 2011; 5: e1309)
ZIKV	4.5	4.7 $\log_{10}$ (n=37; Waggoner et al., Clin Infect Dis 2016; 63: 1584–1590)<br>6.0 $\log_{10}$ (at 9th days post symptom onset, n=57; Musso et al., J Med Virol 2017; 89: 1505–1510)<br>1.07 × 10^5 (in children, n=317; Read et al., JAMA Pediatr 2018; 172: 686–693)	11.5 (n=30; Barzon et al., Clin Infect Dis 2018; 66: 1173–1180)<br>14 (n=150; Paz-Bailey et al., N Engl J Med 2018; 379: 1234–1243)
WNV	not available	not available	not available
HCV	4.3	3.2-6.8 $\log_{10}$ as indicated in 24 references. One example with the largest n: 5.23 $\log_{10}$ (n=548; Alves et al., Braz J Infect Dis 2018; 22: 123–128)	108 (spontaneous clearance, n=17); the other patients (n=95) cannot clear the virus spontaneously (Thomson et al., Gut 2011; 60: 837–845)<br>19 (after antiviral treatment, n=38; Smith et al., Semin

			Thorac Cardiovasc Surg 2020; doi:10.1053/j.semtcvs.2020.06.045)<br>8 patients cleared HCV spontaneously within 34.7 days and the other patients (n=4) showed HCV-RNA negative after 9.0 months (Hofer et al., Hepatology 2003; 37: 60–64)
HBV	0.89	3.0-8.7 $\log_{10}$ as indicated in 23 references. One example with the largest n: 3.2 $\log_{10}$ (n=1876; Bensalem et al., Arch Virol 2017; 162: 1641–1648)	> 28 (n=50; Nowak et al., Proc Natl Acad Sci USA 1996; 93: 4398–4402)<br>79 patients with acute self-limiting hepatitis cleared HBV spontaneously within 42 days, but most chronic patients (16/17) still presented HBV-RNA after 6.0 months (Moestrup et al., J Med Virol 1985; 17: 337–344)
EV71	0.5 (encephalitis);<br>0.3 (no encephalitis)	4.76 $\log_{10}$ (n=317; Huang et al., PLoS One 2016; 11: e0148907)	25 (mild, n=9); 35 (severe, n=14; Li et al., Virol J 2013; 10: 31)<br>24 (n=34; han et al., BMC Infect Dis 2010; 10: 178)

HAART, highly active antiretroviral therapy.

Table S4. IFN-γ Fold-changes, viral load and clearance rate of different virus infections.

Virus	IFN-γ fold-change	Log_{10} Viral load	Clearance time (d)
SARS	6	5.8	21
MERS	6	6.7	19
Non-ICU of SARS-CoV-2	7	6.4	14
ICU of SARS-CoV-2	8	6.4	21
2009H1N1	1.3	6.84	5
H3N2	1.14	6.94	5.14
H7N9 severe	9.3	7.07	19.7
H5N1	6	5.9	27
Ebola survivors	23.3	7.6	158
Ebola fatalities	40	9.5	∞ (infinity)
Dengue fever	8.9	7.7	12
Dengue hemorrhagic fever	22.2	9.0	» 12
ZIKV	4.5	6.0	14
WNV	20	N.A.	N.A.
HIV before HAART	0.16	4.42	31
HIV 1 yr after HAART	0.54	< 4.0	N.A.
HBV	0.89	3.2	> 180
HCV	4.3	5.23	> 270
EV71 with encephalitis	0.5	4.76	35
EV71 without encephalitis	0.3	4.76	25

N.A., not available.

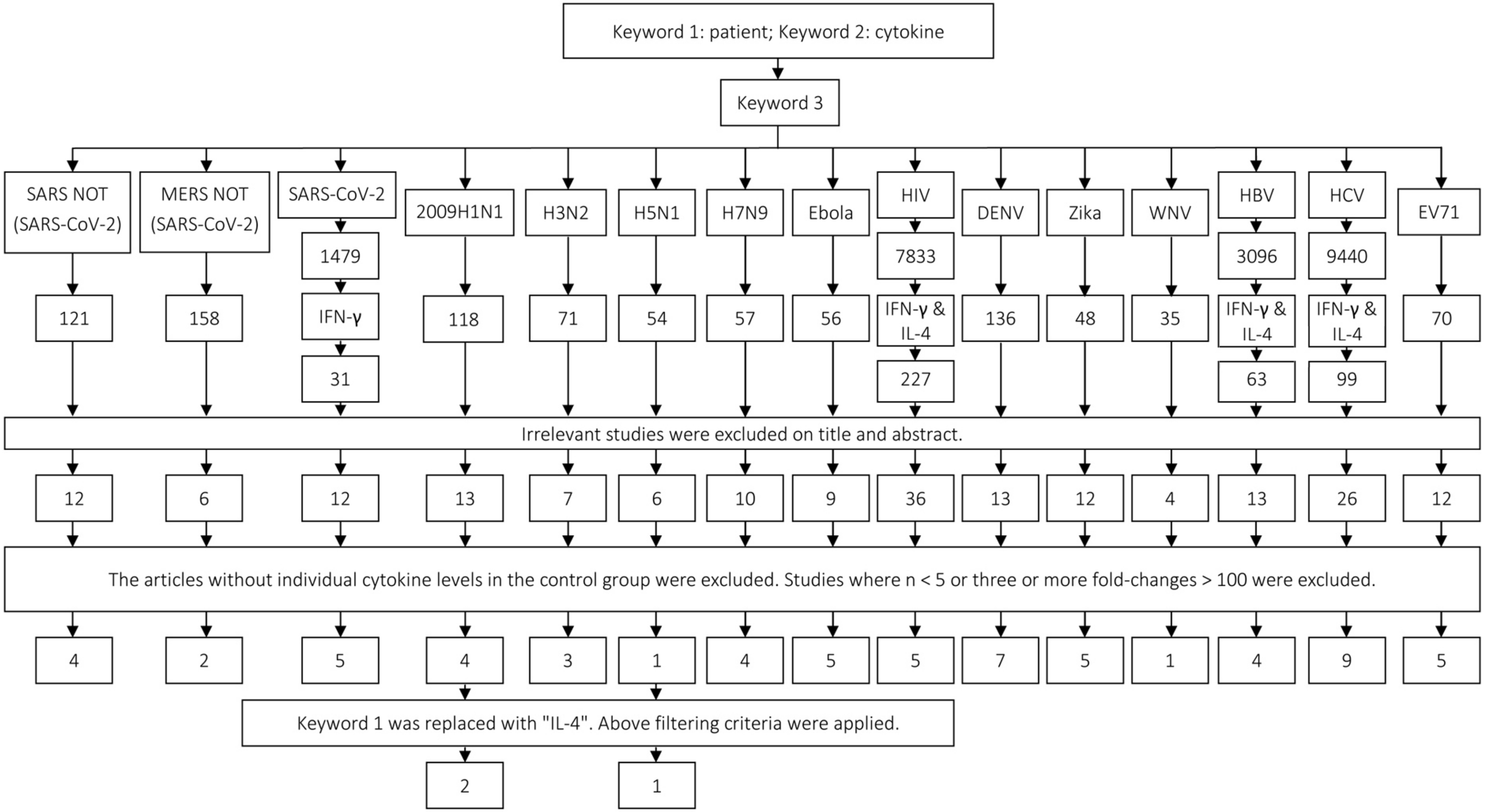

Figure S1. Flow chart algorithm for the literature search to get cytokine change information.

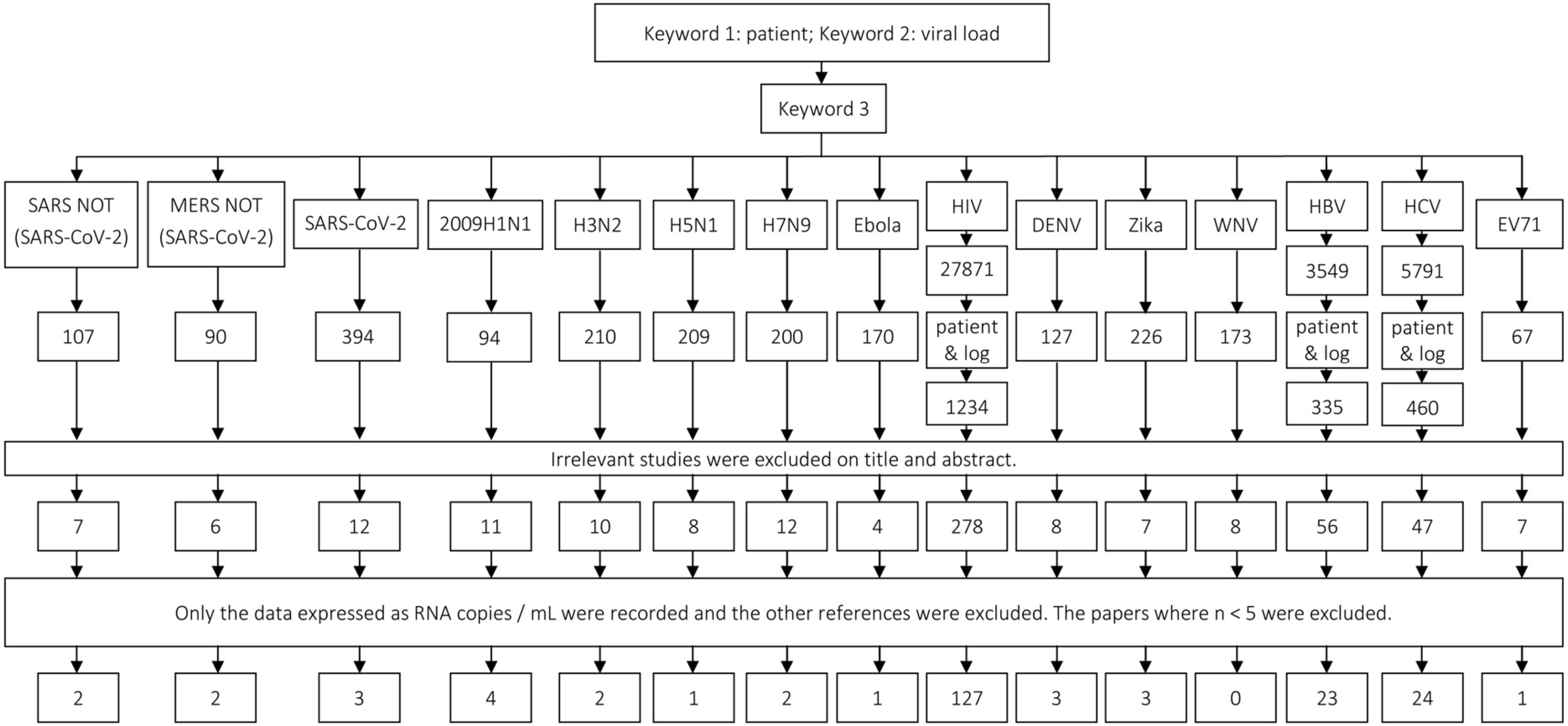

Figure S2. Flow chart algorithm for the literature search to get viral load information.

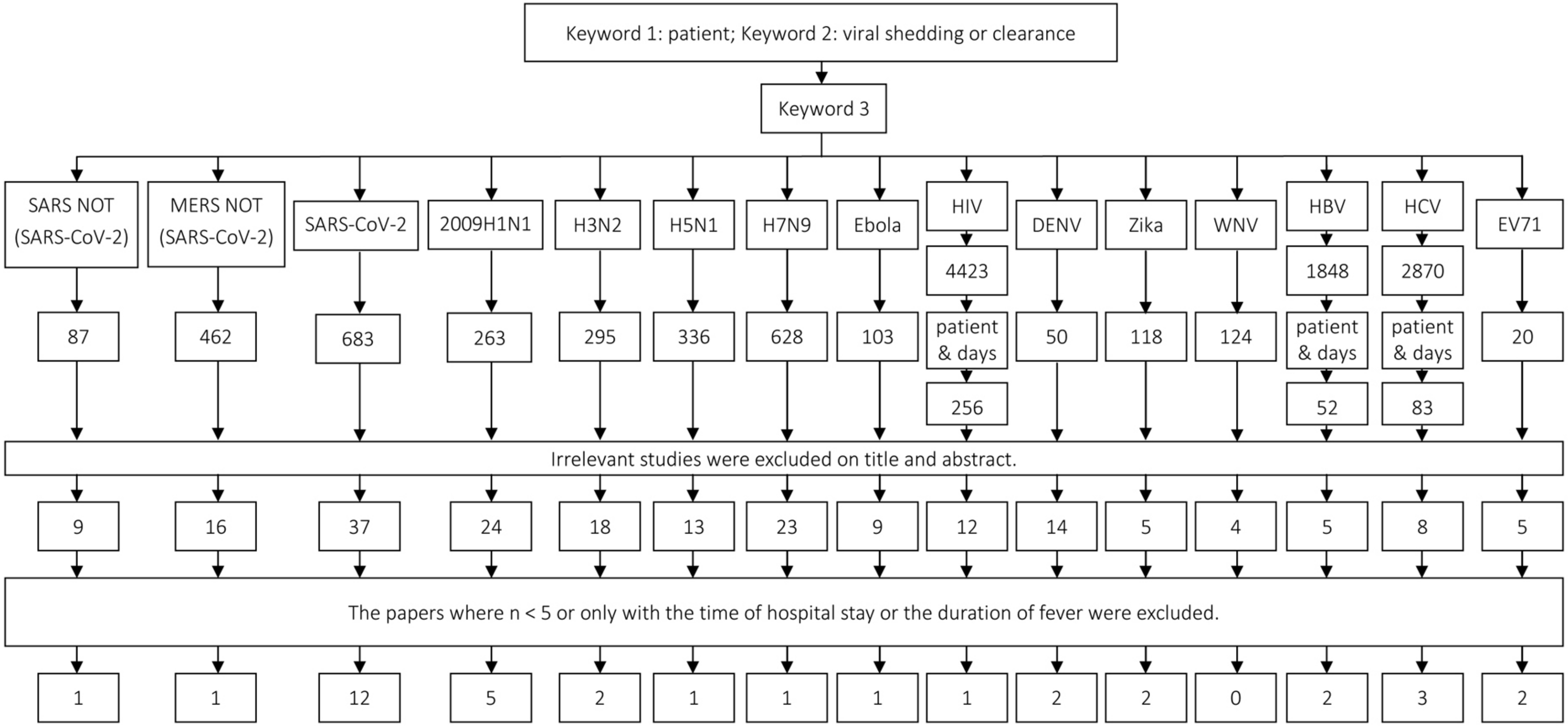

Figure S3. Flow chart algorithm for the literature search to get virus clearance time information.

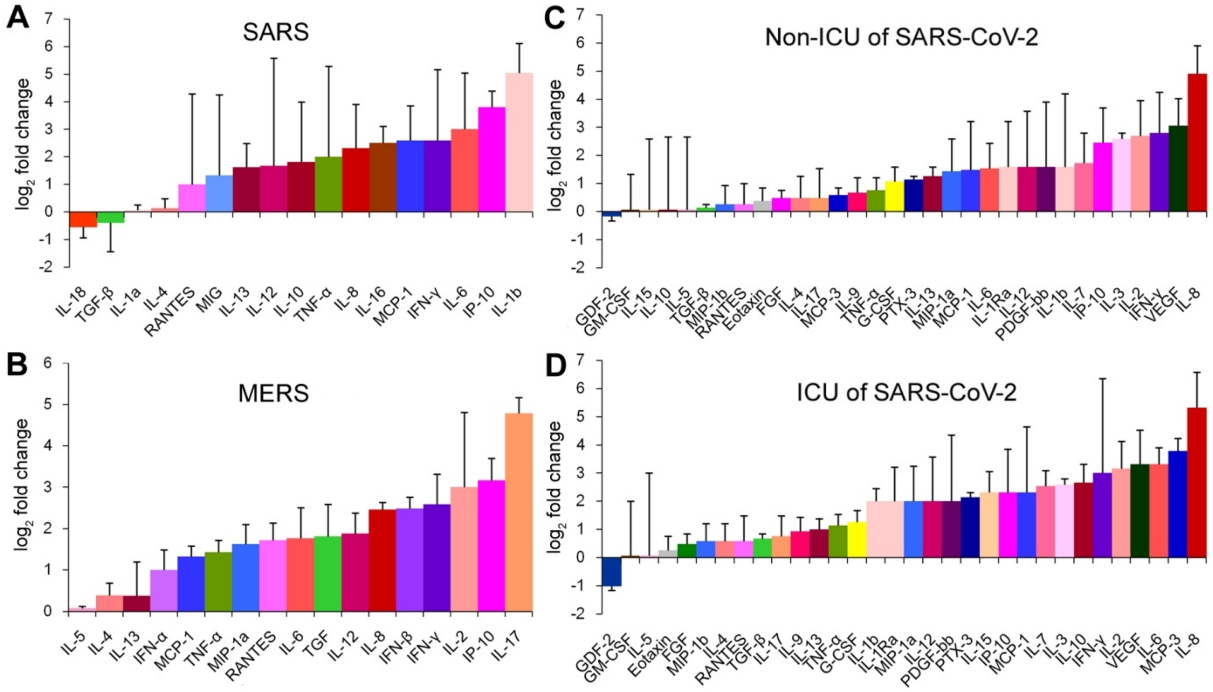

Figure S4. Cytokine changing profile during coronavirus infections. ICU, intensive care unit. Bars show SD, SEM or interquartile ranges as indicated in Table S1.

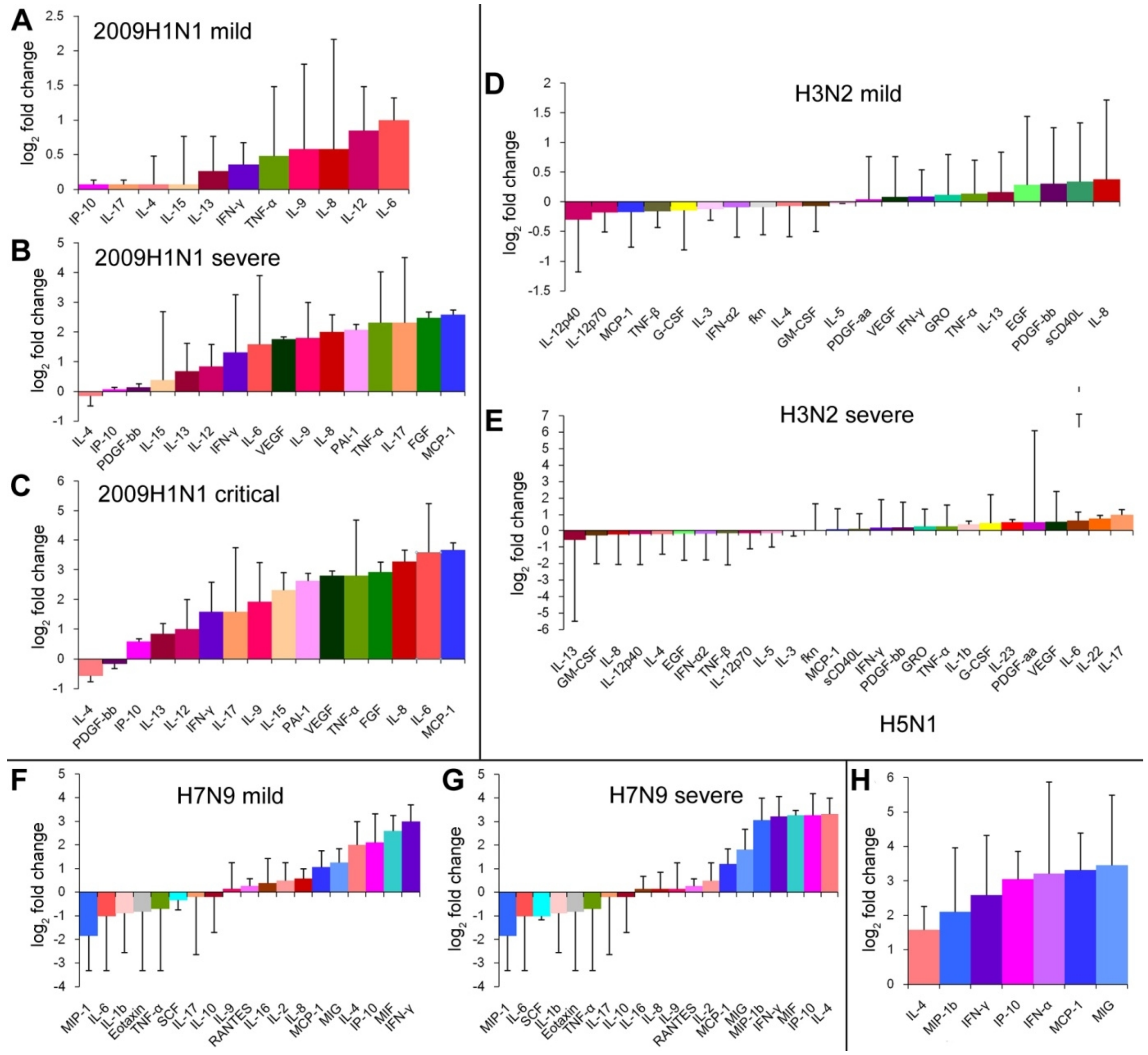

Figure S5. Cytokine changing profile during influenza virus infections. Bars show SD, SEM, interquartile ranges or 95% confidence-intervals as indicated in Table S1.

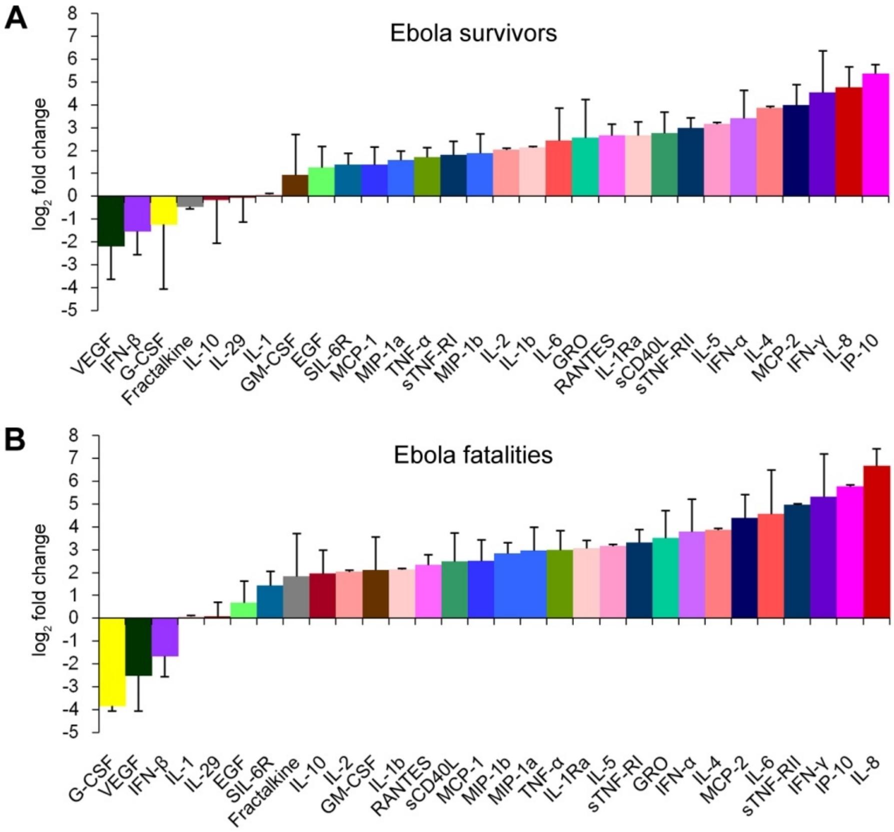

Figure S6. Cytokine changing profile during Ebola virus infections. Bars show SD, SEM, interquartile ranges or the 10th and 90th percentiles as indicated in Table S1.

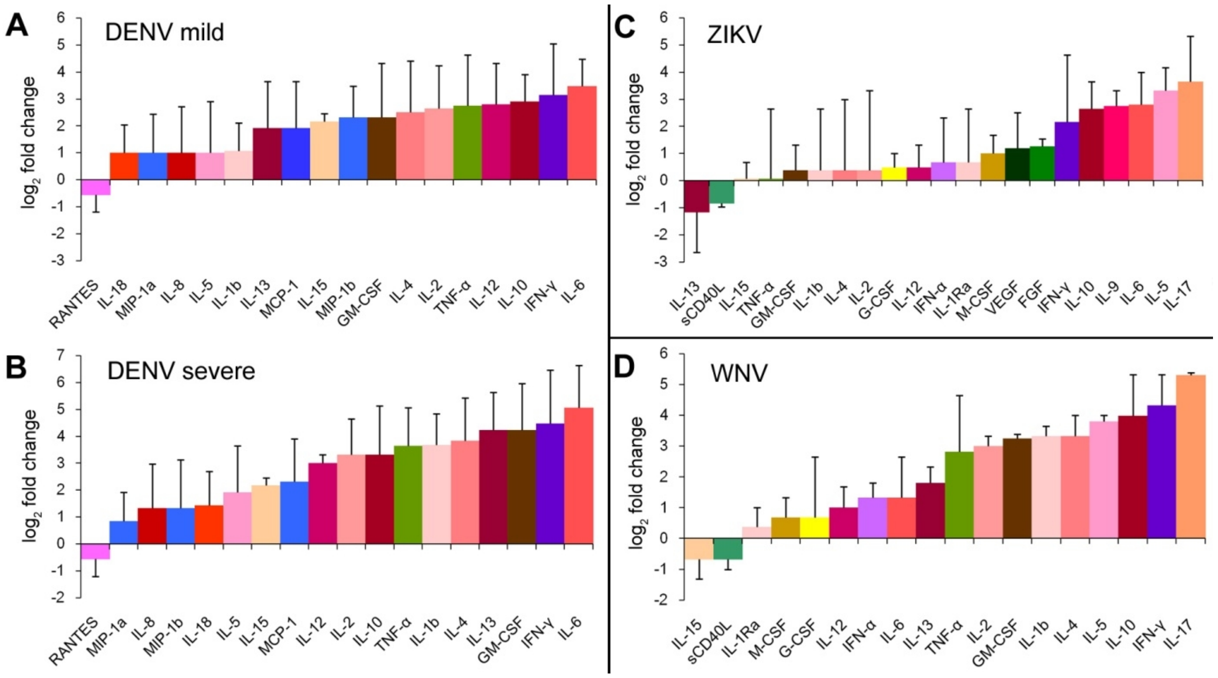

Figure S7. Cytokine changing profile during Dengue virus (DENV), Zika virus (ZIKV), or West Nile virus (WNV) infections. Bars show SD, SEM, interquartile ranges or 95% confidence intervals as indicated in Table S1.

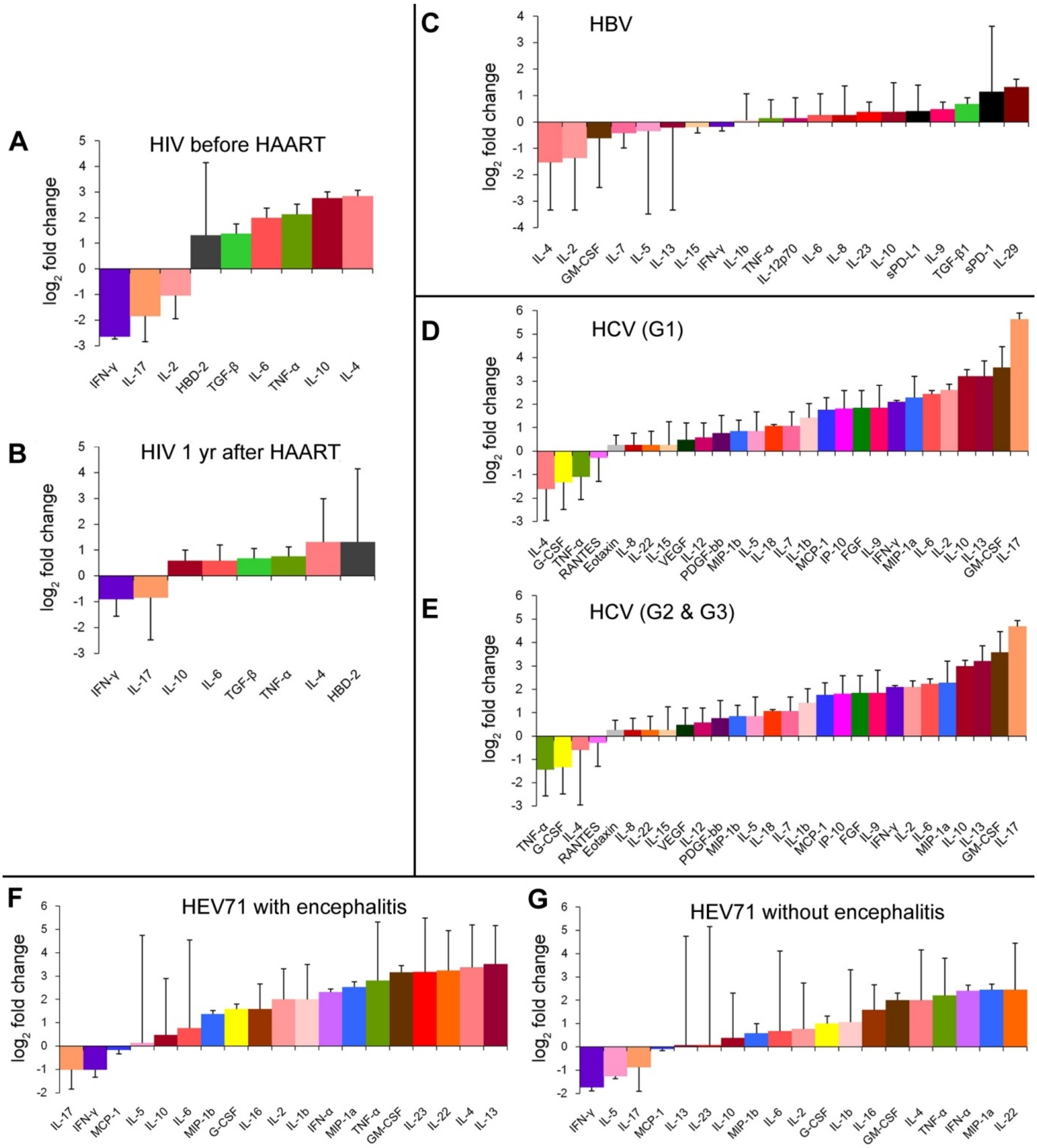

Figure S8. Cytokine changing profile during Human Immunodeficiency Virus (HIV), Hepatitis B virus (HBV), Hepatitis C virus (HCV) or enterovirus 71 (EV71) infections. HAART, highly active antiretroviral therapy. Bars show SD, SEM, interquartile ranges or 95% confidence intervals as indicated in Table S1.
